# The Metropolitan Asylums Board: Some Lost Opportunities

**Published:** 1904-08-20

**Authors:** 


### The Metropolitan Asylums Board: Some Lost Opportunities.

With the plentiful material at their disposal, and the special facilities which exist for collating and analysing it, the Metropolitan Asylums Board have a unique opportunity to record from year to year the exact measure of success and failure attending the sanitary and therapeutic principles which are practised in their institutions; and it can be readily appreciated how necessary it is that there should appear in their annual reports, in addition to the bare statistics, official medical comments wherever the figures show any marked discrepancies from those of former years. By such a course the experience gained in new or modified methods would be rendered available to medical men throughout the country. Happily in the past the medical supplement of the Board's annual report has been a document from which the medical profession as a whole has derived much valuable guidance. It is with some disappointment, therefore, that we find in the recently issued report for 1903 an almost entire absence of medical comment, notwithstanding that there are several points in the statistical tables which might be explained with advantage; and this is the more striking because the supplement is clearly intended for medical readers.

It is to be noted in Table I. of the supplement that, among 10,993 cases of scarlet fever, diphtheria occurred as a complication in 256 as compared with 424 cases last year, while the percentage cases of post-diphtherial scarlet fever dropped from 5·33 in 1902 to 4·44 last year. These reductions are remarkable, and call for more than a passing notice. It was announced in last year's report that attempts to reduce the incidence of diphtheria in scarlet fever cases, and of scarlet fever in diphtheria patients, by accommodating the two illnesses in different institutions, had failed. In view of this pronouncement some explanation of last year's success, which resulted in the saving of many lives, is desirable. Again, in Table VII., which contains a summary of the antitoxin treatment of diphtheria, it is to be observed that out of 530 cases of diphtheria admitted to the North-Western Hospital, 172 were treated without antitoxin and they all recovered, while of the remaining 358 treated with antitoxin 42 died; and of the entire 5,422 diphtheria cases treated in the Board's Hospitals, 4,839 were treated with antitoxin, of whom 10·18 per cent. died, whereas of the 583 cases treated without antitoxin only 1·88 per cent. ended fatally, and an appended note explains away this small percentage by stating that the cases were admitted too late. We may suppose that the instances in which antitoxin was withheld were mild cases of diphtheria; but, ignoring altogether the effect such statistics in their nakedness are likely to have on the anti-antitoxin propagandists, most medical men would like to be informed of the exact criteria by which the Board's medical officials were guided in their decisions whether to administer or to withhold antitoxin.

The tables, also, which deal with enteric fever cases, call for criticism, and especially in regard to the mortality from perforation of the intestine. It appears that this complication occurred 46 times in all. In 19 of these laparotomy was performed, with only one recovery, a percentage mortality for cases operated on of 94·74, and for all cases of perforation of 97·83. These results seem to show room for improvement, and we would suggest that they might be materially improved if surgeons with a wide experience in abdominal surgery were called in to deal with cases of perforation. We do not believe the expense would be prohibitory, and, seeing that to a large extent it is in the interest of the general public that patients suffering from infectious diseases are removed to the isolation hospitals, they are entitled to receive all the care that modern medicine and surgery can provide; and abdominal surgery is at the present time of such a special character that it seems scarcely appropriate to call upon the medical officers of the Board to undertake the responsibilities of laparotomy in these cases. In any event the experiment of calling in specialists would be worth a trial, and if it were found that lives were saved by this course—and we feel little doubt that such would prove to be the case—the additional expenditure would surely be more than justified.

